# Cutaneous Pseudolymphoma As a Rare Adverse Effect of Medicinal Leech Therapy: A Case Report and Review of the Literature

**DOI:** 10.7759/cureus.7517

**Published:** 2020-04-02

**Authors:** Mozhdeh Sepaskhah, Nazafarin Yazdanpanah, Fatemeh Sari Aslani, Mojgan Akbarzadeh Jahromi

**Affiliations:** 1 Molecular Dermatology Research Center, Shiraz University of Medical Sciences, Shiraz, IRN; 2 Dermatology, Shiraz University of Medical Sciences, Shiraz, IRN; 3 Dermatology, Molecular Dermatology Research Center, Shiraz University of Medical Sciences, Shiraz, IRN; 4 Pathology, Shiraz University of Medical Sciences, Shiraz, IRN

**Keywords:** pseudolymphoma, leeching, skin, adverse effects, leeches, hirudo medicinalis, reactive lymphoid hyperplasia

## Abstract

Hirudotherapy (leech therapy) is one of the oldest practices in medical history, and nowadays it is used for several purposes in medicine. Salvage of flaps, wound healing, pain management, and treatment of varicose veins are among the common therapeutic applications of leeches. Complications associated with leech therapy include infections, bleeding, anemia, and allergic reaction. Cutaneous pseudolymphoma (benign proliferation of lymphoid cells in the skin) follows several underlying conditions. Although persistent arthropod bite reaction is one of the conditions associated with cutaneous pseudolymphoma, it has been rarely reported after medicinal leech therapy. Here we describe the case of a patient who presented with cutaneous pseudolymphoma after leech therapy as a rare cutaneous complication of hirudotherapy.

## Introduction

Hirudotherapy (medical leech therapy) is among the first procedures performed by ancient physicians. Leech therapy has been considered as an attractive treatment in several medical fields in recent years. The medicinal leech therapy is mostly used in modern medicine such as plastic and reconstructive surgery for flap salvage, hematoma decompression, post-phlebitic syndrome, and healing of chronic wounds [1]. Despite several advantages, some adverse effects have also been reported, including uncontrolled bleeding and infections associated with gram-negative, aerobic Aeromonas species, which are sensitive to aminoglycosides, fluoroquinolones, and trimethoprim-sulfamethoxazole [1,2].

Cutaneous pseudolymphoma is a benign lymphoid infiltration caused in reaction to a variety of conditions including arthropod bites [3]. Pseudolymphomatous reaction to leech bites was first described by Smolle et al. in 2000 [4]. To the best of our knowledge, English language studies have presented seven other cases so far [5-11]. Herein, a new case of cutaneous pseudolymphoma is presented at the site of medicinal leech therapy.

## Case presentation

**Figure 1:**
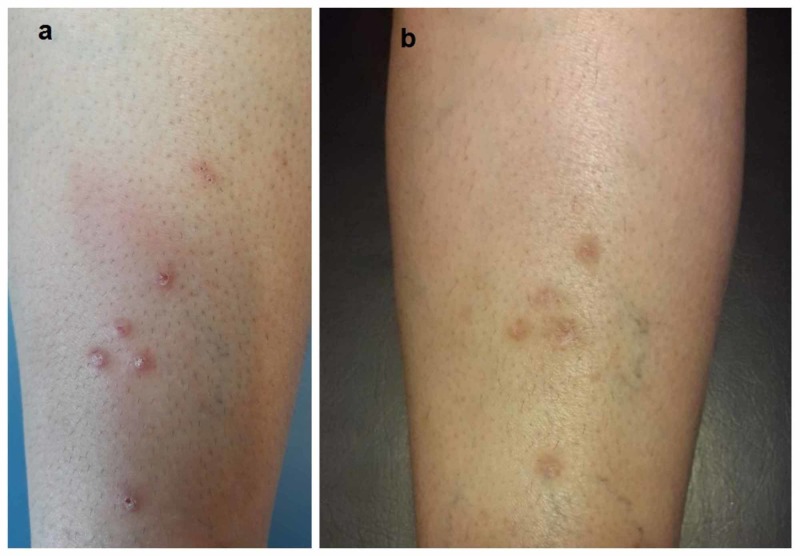
Clinical features. (a) Pruritic crusted erythematous papules on the background of erythematous, tender, and warm subcutaneous nodules. (b) Mildly erythematous and hyperpigmented macules on the site of leech bites after treatment

Our case was a 44-year-old woman referred to the rheumatologist with multiple painful and erythematous pruritic lesions on her right shin 1.5 months after leech therapy prescribed by a general practitioner for the treatment of erythema nodosum (EN) that developed four months before referral on her right shin. According to physical examination of the skin, there were seven pruritic crusted erythematous papules on the background of erythematous, tender, and warm subcutaneous nodules on the right shin (Figure 1a).

The laboratory workups including complete blood count test, liver function test, and tests for the assessment of thyroid-stimulating hormone, blood calcium and phosphorus, antinuclear antibody, double-stranded DNA, lactate dehydrogenase, and angiotensin-converting enzyme were in the normal range. However, erythrocyte sedimentation rate (29 mm/h; normal range: 0-20 mm/h) and C-reactive protein (7.8 mg/L; normal: <5mg/L) were mildly elevated. Skin biopsies were performed for both subcutaneous nodules and crusted papules. Administration of oral prednisolone 40 mg/kg/day was started and gradually tapered to be discontinued according to the clinical diagnosis of EN (tender and erythematous nodules on the shin due to septal panniculitis) by the rheumatologist.

**Figure 2:**
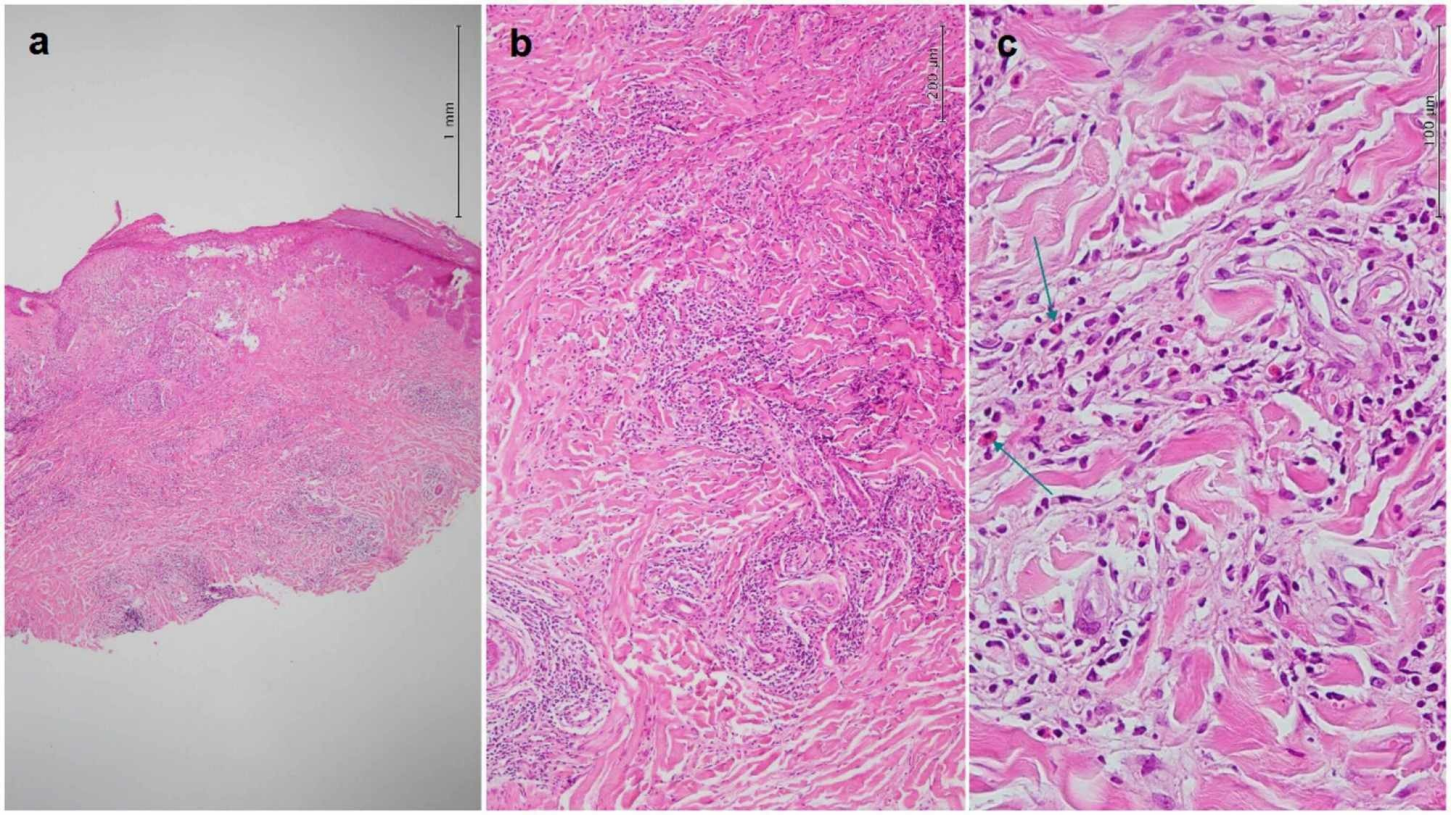
Histopathological features. (a) Hyperkeratosis, crust formation, mild acanthosis, focal spongiosis, and exocytosis of lymphocytes. Dense perivascular inflammatory infiltration of superficial and deep (hematoxylin and eosin, 40×). (b) Superficial and deep dermal dense perivascular infiltration of mixed populations of lymphocytes, histiocytes, and eosinophils (hematoxylin and eosin, 100×). (c) Dense perivascular infiltration of mixed populations of lymphocytes, histiocytes, and eosinophils (arrows) around the vessels (hematoxylin and eosin, 400×)

Histological examination of the skin biopsy samples showed hyperkeratosis, crust formation, mild acanthosis, focal spongiosis, and exocytosis of lymphocytes. Superficial and deep dermal infiltrate mainly with lymphocytes was observed, admixed with some histiocytes, eosinophils, and plasma cells around vessels (Figure 2a-c). Despite the presence of a clinically typical EN lesion, the biopsy of subcutaneous nodules was superficial and had an inconclusive pathological diagnosis.

This mixed cellular infiltrate was in favor of pseudolymphoma. Colocalization of the skin lesions with mentioned histopathology to the site of the previous leech therapy confirmed the diagnosis of leech-induced pseudolymphoma. The patient could not afford immunohistochemistry.

The patient was taking oral prednisolone for the treatment of EN when the pseudolymphoma was diagnosed, which also resulted in the improvement of pseudolymphoma. During the one-month follow-up, the papules were resolved, leaving mild erythema and postinflammatory hyperpigmentation (Figure 1b). Therefore, we added mometasone ointment to be applied once daily. The lesions did not recur during the six-month follow-up.

## Discussion

Hirudotherapy mechanism is based on the secretion of biologically active substances from the salivary gland of leeches. Secretions from salivary glands of leeches contain anti-inflammatory, bacteriostatic, and anticoagulant factors [1]. Nowadays, leech therapy is effectively used by surgeons for venous disorders, surviving necrotic flaps, reattachment surgery, resolving hematoma, and chronic wounds [1]. Pain management, cardiovascular diseases, metastasis of cancers, diabetic complications, and arthritis are among the other conditions treated by leech therapy in modern medicine [2]. In spite of advantages, some side effects have also been reported for leech therapy. Infection is the most common reported adverse effect of leech therapy. Bacterial infection in the leech intestine, including Aeromonas species, is a common complication requiring prophylactic antibiotic therapy [1,2]. The bacteria and leech are symbiotic; therefore, even medical leeches are not organism-free. Several approaches that are offered to restrict infectivity of leeches are not ideally beneficial [12]. The patients may experience prolonged bleeding and anemia during leech therapy [1]. Pruritus, vesicle formation, necrotic ulcer, and allergic and irritant contact dermatitis have been reported as cutaneous complications of medicinal leech therapy [1,2]. Smolle et al. reported the first case of cutaneous pseudolymphoma as a rare complication of medicinal leech therapy [4].

Cutaneous pseudolymphoma is defined as a benign proliferation of lymphoid cells in the skin caused by several underlying conditions that mimic lymphoma clinically and/or histologically [3]. Persistent arthropod bite reaction is among the conditions associated with the development of cutaneous pseudolymphoma. Histological features of this condition include dense perivascular superficial and deep dermal lymphocytic (mostly T cell) infiltrate, admixed with other inflammatory cells such as eosinophils and plasma cells [3]. Persistent bite reaction frequently occurs following scabies and less commonly other arthropod bites. Few cases of pseudolymphomatous bite reaction have been reported after medicinal leech therapy [5-11]. Table 1 summarizes the features of the cases of cutaneous pseudolymphoma after medicinal leech therapy reported in the English language studies indexed in PubMed and Google Scholar.

 

**Table 1:** Clinicopathological features of leech-induced pseudolymphoma cases in the literature MLT, medicinal leech therapy; F, female; N/A, not available; IL, intralesional; CS, corticosteroid; M, male; IM, intramuscular

Article	Age (years)	Gender	Site	Time lapse after MLT	Reason for MLT	Lesion type	Symptom	Leech-applying person (or institute)	Prominent cell type	Clonality	Treatment
Smolle et al 2000	56	F	Lower leg	Several weeks	Venous insufficiency	Nodules Scar	N/A	N/A	B cell	Polyclonal	IL CS
Choi and Kim 2012	52	M	Lower eyelids	Several months	Dark circles	Nodules	N/A	Non-medical personnel	T cell	N/A	IL CS
Khelifa et al 2013	77	F	Lower back	Several months	Low back pain	Nodules	Asymptomatic	Private, alternative medicine clinic	Mixed B and T cells	Polyclonal	Topical CS, IL CS
Altamura et al 2014	50	F	Back	5-6 weeks	Fibromyalgia	Nodules Papules	Pruritus	N/A	B cells	N/A	Topical CS
Tupikowska et al 2018	38	F	Pubis	N/A	Uterine myoma	Nodules	Pruritus	A friend	Mixed B and T cells	N/A	IL, topical, IM CS, cryotherapy
Aktaş et al 2018	65	F	Lower back	A few months	Lumbar pain	Nodules	Pruritus	N/A	N/A	Polyclonal	IL CS, cryotherapy
Temiz et al 2019	54	M	Neck	3-5 months	N/A	Plaques	Pruritus	N/A	T cell	N/A	IL CS
Sadati et al 2019	45	F	Leg	1 month	Varicosities	Papules	Pruritus	General practitioner	N/A	N/A	Topical CS, cryotherapy
Present case	44	F	Leg	1.5 months	Erythema nodosum	Papules	Pruritus	General practitioner	N/A	N/A	IL, topical, oral CS

Most of the reported cases of leech-induced pseudolymphoma have been female patients [4,6-9,11]. Most of the cases have tried it as a treatment for a medical problem including venous insufficiency, back pain, uterine myoma, and fibromyalgia [4,6-9,11]. Only one patient experienced leech therapy to relieve a cosmetic problem (periocular dark circle). The leech was applied by a non-medical personnel in this case [5]. Our patient was advised to try leech therapy for the treatment of EN. Site of the lesions depends on the site of underlying problem treated by a leech. Previous studies have reported various time lapses between leech therapy and the appearance of pseudolymphoma, including several weeks to several months [4-7,10,11]. Pseudolymphoma at the site of leech therapy is mostly presented in the form of firm, erythematous, brown or purple nodules, or papules; however, the lesions are rarely presented as plaques [[4-9,7,11,10]. Some studies reported pruritus as a symptom of pseudolymphoma following leech therapy [7-11]. In the case of our patient, the lesions were pruritic papules. Scar formation has been mentioned in one case [4].

Histologically, most of the cases presented superficial and deep dermal infiltrate of mostly lymphocytes, admixed with other inflammatory cells including eosinophils, plasma cells, and histiocytes, which is similar to the histopathology of our patient [4,6-11]. In one case, the infiltrate was mostly in the upper dermis [5]. Germinal centers have been found in a few cases [4,7]. There is an inconsistency in previous studies regarding epidermal changes including hyperkeratosis, acanthosis, focal spongiosis, and exocytosis of intraepidermal inflammatory cells [8,10]. Epidermal changes were prominent in our case. B cell, T cell, or mixtures of B and T cells have been found to be predominant in immunohistochemistry [4-8,10,11]. The lymphocyte population is polyclonal [4,6,9]. Scar is a rare histological feature of pseudolymphoma [4].

Different management approaches are applied to treat pseudolymphoma following leech therapy including potent and superpotent topical corticosteroids, intralesional and systemic corticosteroids, and cryotherapy, which are generally beneficial [4-11]. Our patient received systemic prednisolone for the underlying EN along with topical mometasone and intralesional triamcinolone, and the lesions were healed with residual mild postinflammatory hyperpigmentation.

## Conclusions

Pseudolymphoma is a rare but emerging side effect of hirudotherapy as a common traditional medicinal treatment in many countries. This diagnosis should be considered in any patient who presents with persistent skin papules and nodules at the site of leech therapy.
